# Knowledge, Attitude, and Practice of Health Workers in a Tertiary Hospital in Ile-Ife, Nigeria, towards Ebola Viral Disease

**DOI:** 10.1155/2015/431317

**Published:** 2015-10-20

**Authors:** Samuel Anu Olowookere, Emmanuel Akintunde Abioye-Kuteyi, Olusegun Kayode Adepoju, Oluwaseun Taiwo Esan, Temitope Michael Adeolu, Tolulope Kola Adeoye, Adesola Adebayo Adepoju, Adedayo Titilayo Aderogba

**Affiliations:** Department of Community Health, College of Health Sciences, Obafemi Awolowo University, Ile-Ife, Nigeria

## Abstract

*Background*. Health workers are more prone to Ebola viral disease (EVD) than the general population. This study assessed the preparedness of health workers in the control and management of EVD. *Methods*. A descriptive cross-sectional study. Consenting 400 health workers completed a semistructured questionnaire that assessed participants' general knowledge, emergency preparedness, and control and management of EVD. Data were analysed using descriptive and inferential statistics. *Results*. The mean age (SD) was 34.5 ± 8.62 years ranging from 20 to 59 years. Most participants were medical doctors (24.6%) and nurses (52.2%). The majority had practised <10 years (73.8%) and were aware of the EVD outbreak in the West African subregion (85.5%). Colleagues (40%) and radio (37.2%) were their major sources of information. Only 42% had good knowledge while 27% knew that there was no vaccine presently to prevent EVD. About one-quarter (24.2%) had low risk perception. The majority (89%) felt the hospital infection control policy was inadequate to protect against EVD. The only predictor of good knowledge was participants' occupation. *Conclusion*. There is knowledge gap and poor infection control preparedness among respondents. Thus, knowledge and practices of health workers towards EVD need improvement.

## 1. Introduction

Ebola viral disease (EVD) is an acute febrile illness caused by the Ebola virus, a member of the family of Filoviridae. EVD is associated with a high mortality rate in humans and non-human primates since its initial recognition in the Democratic Republic of the Congo in 1976 [1, 2]. The Filoviruses are thread-like RNA viruses that cause haemorrhagic fever. The Ebola virus causes severe disease in humans with an extremely high case fatality rate ranging from 25 to 90%, depending on the viral subtype and the availability of medical care. Haemorrhagic symptoms occur in about 30–50% of described human cases [1]. Four Ebola viral subtypes (Zaire, Sudan, Ivory Coast, and Uganda) and the Marburg virus cause illness in humans, and the subtypes Zaire and Ivory Coast and the Marburg virus are known to cause illness in non-human primates. One Ebola subtype (Reston) causes illness in non-human primates but has induced only asymptomatic disease in humans [3]. The natural reservoir of the Ebola viruses remains unknown. The incubation period of Ebola virus is 2–21 days and it is transmitted majorly through direct contact with body fluid including blood, urine, excreta, vomit, saliva, sweat, mother's breast milk, organs, body parts, secretions, and seminal fluid. A rare mode of transmission is contact with the unknown natural reservoir or infected animals. Routes of infection are oral, the conjunctivae, mucous-membrane exposure (e.g., nose and mouth), sexual intercourse, and a break in the skin, a penetrating object infected with body fluids of a patient (e.g., needles or razor blades). Infections occur when health staff or relatives are taking care of a patient without proper protection. Contact with infected corpses (human or animal) put people at high risk to become infected with EVD. Nosocomial transmissions of EVD do occur when appropriate precautions are not taken [1].

The latest outbreak which affects Guinea, Liberia, Sierra Leone, and Nigeria in West Africa is the worst in EVD history with 2127 reported cases out of which 1145 died by August 15, 2014 [4]. Health workers were included among the infected and the dead from EVD while caring for people infected with this highly fatal disease. EVD prevention and control in the region raises a number of challenges for healthcare workers practising in countries where health systems and infrastructures are weak; healthcare financing is poor and health insurance coverage is limited [5]. Very few studies on EVD had been conducted among health workers in Nigeria [6]. It is therefore imperative to assess the preparedness of health workers in the control and management of EVD.

## 2. Methods

The study was conducted at the Obafemi Awolowo University Teaching Hospital Complex (OAUTHC), Ile-Ife, Nigeria, in the month of July, 2014. It is a 576-bedded hospital with referrals from neighbouring states such as Oyo, Ondo, Ekiti, Kogi, Kwara, and beyond.

The study population included the clinical members of staff, namely, medical doctors, nurses, pharmacists, medical laboratory technologists, community health workers (CHEW), medical records officer, and physiotherapists.

The sample size of 352 was calculated using an appropriate statistical formula for estimating the minimum sample size in descriptive health studies [*n* = *Z* ^2^ *pq*/*d* ^2^] [7], where 64.4% of health care workers knew that EVD had no cure [8]. A sample size of 400 was used after nonresponders being taken into consideration.

The number allocated to each group of clinical staff was determined proportionately using the formula *n*/*N* × 400, where *n* is the number of occupational groups and *N* is the total number of clinical staff [9].

Consenting health workers completed a pretested semistructured self-administered questionnaire that assessed participants' general knowledge, emergency preparedness, and control and management of EVD. The questionnaires were distributed consecutively to members of each occupational group during the break period. The respondents were allowed to fill the questionnaire in their spare time at their convenience. Questionnaire information was anonymised.

Ethical approval to conduct the study was obtained from Ife Central Local Government Ethical Review Committee. Written informed consent was taken from the respondents while they were reassured of the confidentiality of the information obtained. The data collected were entered and kept in a password protected computer.

The data obtained were analysed using SPSS version 16. Simple descriptive and inferential statistics were done. Knowledge score was computed for a 41-item question on knowledge of EVD. Each item was assigned “+1” for correct knowledge and “0” for incorrect knowledge. The knowledge score was graded as good or appropriate (if respondent scored ≥ 27 points) and not good or not appropriate (if score was <27 points) using the mean score as the break-off point. Test of significance was conducted using appropriate statistical methods. Multivariate analysis was performed using logistic regression to evaluate sociodemographic variables and other variables that are independently associated with good knowledge of EVD. Adjusted odd ratio (AOR) and 95% CI were presented and used as measures of the strength of association. Significant level was put at *p* < 0.05.

## 3. Results

Four hundred completed questionnaires were analysed. The mean age (SD) of the respondents was 34.5 ± 8.62 years (range 20–59 years). Most participants were medical doctors (24.6%) and nurses (52.2%). The majority were females (60.2%), were married (65.8%), and had practised <10 years (73.8%) (Table 1).

The majority 342 (85.5%) were aware of the on-going EVD outbreak in the West African subregion. Colleagues (40%) and radio (37.2%) were their major sources of information (Table 2).

**Figure 1:**
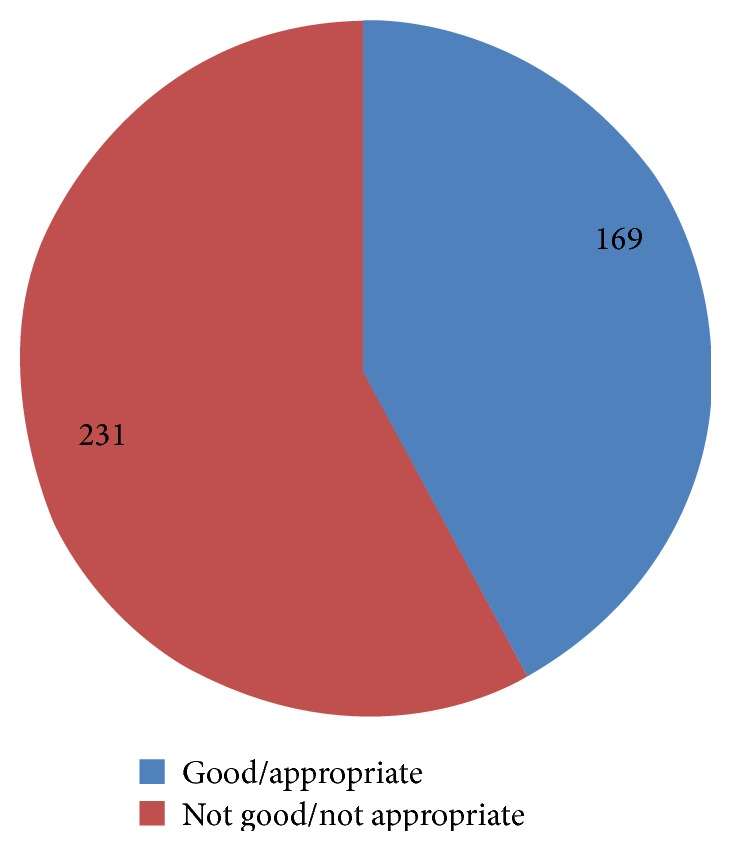
Graded score on EVD knowledge.

Only 42.3% had good knowledge of EVD (Figure 1). Most knew that EVD is a viral infection (93.2%) that is deadly (91.5%). Also, majority knew that EVD can be transmitted from person to person (87.8%) and animal to person (86.2%) while only 46.8% knew it can be transmitted from inanimate objects to persons (Table 3).

Although the majority of participants knew that EVD is transmissible through body fluids, below half knew that the causative agent penetrates broken skin (Table 4).

Most health workers knew high grade fever (78.2%) and unexplained bleeding (73.4%) as common presentation in EVD patients while fewer health workers knew that gastrointestinal symptoms and shock (27%) could occur in these patients (Table 5).

Although majority of participants knew some treatment and prevention of EVD, about three-quarter did not know that EVD has no vaccine presently (Table 6). Most respondents were not aware of the process for EVD reporting (Table 7). Most respondents had poor risk perception and negative attitude to EVD diagnosis, management, and prevention. About 11% felt that the infection control policy of the hospital is adequate to protect health workers against EVD (Table 8).

Medical doctors (54.1%) and nurses (42.6%) had appropriate knowledge compared to other health workers and this association was statistically significant (Table 9).

The only predictor of good/appropriate knowledge was participants' occupation (Table 10).

## 4. Discussion

This study assessed the knowledge, attitude, and practice of health workers in a tertiary hospital in the south-western part of Nigeria towards EVD. The health workers that participated included medical doctors, nurses, pharmacists, medical laboratory technologists, medical record officers, physiotherapists, and community health workers. All these health workers come into contact with patients or their body fluids in the work place. Hence this baseline study determines their preparedness towards EVD. Most participants were young, were females, were married, and had practised less than 10 years. This implies that the participants still had more years to work and hence the necessity to remain healthy in order to perform their health care duties. Also, these health workers could be a source of spread of this life threatening infection to coworkers, their families, and community.

Although most respondents were aware of the EVD epidemic in the West African subregion, some were not aware. This is not acceptable as every health worker should be aware to ensure necessary precautions are taken to reduce the ongoing epidemic and control it whenever such spreads to the facility. This will ensure early diagnosis, management, control, and reporting of such cases to appropriate authority whenever they occur. Colleagues and radio were identified by the participants as their major sources of information. This shows the importance of peers and media in information management. Studies in Nigeria and elsewhere had reported the radio as a valid means of spreading current information to hospital workers as well as the general populace [8, 9].

Over half of the respondents had poor knowledge of EVD. This is probably because as at the time this study commenced, no case was reported in Nigeria. EVD was seen by most respondents as too far away to be a problem. However, this perception about a dangerous infectious disease such as EVD could result in uncontrollable epidemics; hence attitudinal change will be necessary if this must be averted. The World Health Organization had reported several cases of EVD outside the epidemic zone with Nigeria reporting its first case on July 20, 2014 [10]. This study reported that most respondents knew that EVD is caused by a deadly virus while they did not know it can penetrate broken skin. Also, most respondents did not know that EVD has no vaccine presently and were not aware of the process for EVD reporting. This shows the gap in knowledge that must be filled urgently.

Most participants felt that the infection control policy of the hospital was inadequate to protect health workers against EVD. This implies that the hospital authorities must do all that is required to develop a policy targeting EVD.

Occupation was found to be the only predictor of EVD knowledge with medical doctors and nurses having better knowledge more than other health workers. This reflects the training undergone by these groups of workers and the need to strengthen the capacity of other health workers with adequate knowledge of preventing, diagnosing, and managing EVD cases. All health workers need continuous education on EVD. Also, the training should focus on the concept of universal precautions, which must be observed by every health care worker while interacting with every patient [11].

This baseline study is limited by its cross-sectional design and the fact that some respondents could have given socially acceptable answers to some questions. However, this study will serve as a guide for planning and implementing interventions targeted at controlling possible epidemics in the study area.

## 5. Conclusion

In conclusion, most health workers had inappropriate knowledge about EVD; hence continuous medical education focusing on the concept of universal precautions should target all health workers. Also, an infection control policy targeting EVD is urgently required and emergency preparedness towards possible EVD epidemic is necessary.

## Figures and Tables

**Table 1:** Sociodemographic characteristics of participants.

Variable	Frequency	%
Age group (years)		
20–29	135	33.8
30–39	164	41
≥40	101	25.2
Sex		
Male	159	39.8
Female	241	60.2
Marital status		
Single	137	34.2
Married	263	65.8
Occupation		
Medical doctor	98	24.6
Nurse	209	52.2
Pharmacist	25	6.3
Medical laboratory technologist	23	5.8
Community health officers	19	4.7
Medical records officer	17	4.2
Physiotherapist	9	2.2
Duration of employment (years)		
<10	295	73.8
≥10	105	26.2

**Table 2:** EVD awareness and source of information on the outbreak.

Variable	Frequency	%
Aware of EVD epidemic in West Africa		
Yes	342	85.5
No	58	14.5
^*∗*^Source of information		
Colleagues	160	40.0
Radio	149	37.2
Internet	114	28.4
Television	93	23.3
Newspapers	92	23.1
Notice boards/pamphlets	44	10.9

^*∗*^Multiple responses.

**Table 3:** EVD knowledge of clinical variables.

Variable	Frequency	%
Ebola fever is a viral disease		
Yes	373	93.2
No	27	6.8
Incubation period last from 2 to 21 days		
Yes	247	61.8
No	153	38.2
The reservoir is usually bats		
Yes	270	67.5
No	130	32.5
Infection with the organism is usually deadly		
Yes	366	91.5
No	34	8.5
Ebola can be transmitted from person to person		
Yes	351	87.8
No	49	12.2
Ebola can be transmitted from animal to person		
Yes	345	86.2
No	55	13.8
Ebola can be transmitted from inanimate objects to person		
Yes	187	46.8
No	213	53.2

**Table 4:** Knowledge of EVD mode of transmission.

Variable	Frequency	%
Ebola can be transmitted through saliva		
Yes	274	68.5
No	126	31.5
Ebola can be transmitted through blood		
Yes	331	82.8
No	69	17.2
Ebola can be transmitted through seminal/vagina fluid		
Yes	222	55.5
No	178	44.5
Causative agent penetrates broken skin		
Yes	186	46.5
No	214	53.5
Bodies of dead cases constitute a potential hazard		
Yes	300	75
No	100	25
Cases cease to be infectious after the acute phase of the disease		
Yes	107	26.8
No	293	73.2

**Table 5:** Knowledge of EVD clinical presentation.

EVD cases are characterized by fever >38°C		
Yes	313	78.2
No	87	21.8
Unexplained bleeding could be diagnostic		
Yes	294	73.4
No	106	26.6
Vomiting, diarrhoea, and shock are rarely observed in hospitalized patients		
Yes	292	73.0
No	108	27.0
Fever refractory to treatment and unexplained mucosal bleeding is a sign		
Yes	296	74.0
No	104	26.0

**Table 6:** EVD knowledge of treatment and prevention.

Variable	Frequency	%
^*∗*^Drug use for treatment		
Antipyretics	343	85.8
IV fluids	339	84.8
Corticosteroids	212	53.0
Uses of vaccine protect from EVD infection		
Yes	292	73.0
No	108	27.0
Environmental sanitation protects from infection		
Yes	350	87.5
No	50	12.5
Safe sex protects from infection		
Yes	248	62.0
No	152	38.0
Barrier nursing protects from infection		
Yes	333	83.2
No	67	16.8
Cases can be confirmed without laboratory assistance		
Yes	125	31.2
No	275	68.8

^*∗*^Multiple responses.

**Table 7:** Knowledge of EVD reporting.

Variable	Frequency	%
Critical number of cases must occur before reporting		
True	174	43.5
False	150	37.5
Not sure	76	19.0
Suspected cases qualify for reporting		
True	322	80.5
False	20	5.0
Not sure	58	14.5
Cases should be reported weekly for administrative efficiency		
True	227	56.8
False	67	16.8
Not sure	106	26.4
^*∗*^Tertiary health facilities should notify directly		
Federal ministry of health	290	72.5
State ministry of health	211	52.8
Local ministry of health	208	52.0

^*∗*^Multiple responses.

**Table 8:** Risk perception and attitude to EVD.

Variable	Frequency	%
Consider self to be at risk		
Agree	156	39.0
Disagree	173	42.8
Undecided	71	18.2
Health workers are prone to having EVD		
Agree	303	75.8
Disagree	51	12.7
Undecided	46	11.5
It is possible to prevent EVD spread		
Agree	318	79.5
Disagree	35	8.7
Undecided	47	11.8
There is no risk in living with EVD patient		
Agree	20	5.0
Disagree	332	83.0
Undecided	48	12.0
Infection control policy of the hospital is inadequate		
Agree	109	27.3
Disagree	42	10.5
Undecided	249	62.2

**Table 9:** Association between respondents' characteristics and knowledge of EVD.

Variable	Knowledge		*χ* ^2^	*p* value
	Appropriate	Not appropriate		
Age (years)				
20–29	58 (43.0)	77 (57.0)	5.720	0.057
30–39	78 (47.6)	86 (52.4)		
≥40	33 (32.7)	68 (67.3)		
Sex				
Male	70 (44.0)	89 (56.0)	0.341	0.559
Female	99 (41.1)	142 (58.9)		
Marital status				
Currently married	111 (42.2)	152 (57.8)	0.001	0.980
Not currently married	58 (42.3)	79 (57.7)		
Occupation				
Medical doctor	53 (54.1)	45 (45.9)	12.291	0.002
Nurse	89 (42.6)	120 (57.4)		
Other health workers	27 (29.0)	66 (71.0)		
Duration of employment (years)				
<10	132 (44.7)	163 (55.3)	2.869	0.090
≥10	37 (35.2)	68 (64.8)		

**Table 10:** Binary logistic regression of respondents' characteristics and EVD knowledge.

Variable	AOR	95% CI	*p* value
Occupation			
Medical doctor	2.879	1.582–5.239	0.001
Nurse	1.813	1.072–3.065	0.026
Other health workers (ref.)	1		
